# Transient Insulin-Induced Edema Following Rapid Metabolic Correction in Poorly Controlled Pediatric Type 1 Diabetes Mellitus

**DOI:** 10.7759/cureus.100769

**Published:** 2026-01-04

**Authors:** Tuqa A Abdulsalam, Sara Ali

**Affiliations:** 1 General Pediatrics, Al Jalila Children's Specialty Hospital, Dubai, ARE; 2 Pediatric Emergency Medicine, Al Jalila Children's Specialty Hospital, Dubai, ARE

**Keywords:** acute weight gain, continuous glucose monitoring, desmopressin, diabetic ketoacidosis, diabetic ketoacidosis (dka), fluid retention, insulin-induced edema, pediatric diabetes, rapid insulin intensification, type 1 diabetes mellitus (t1dm)

## Abstract

Insulin-associated edema is an uncommon but well-documented side effect of insulin therapy. It typically occurs with the initiation or rapid escalation in dosage of insulin, especially in patients who have experienced prolonged uncontrolled hyperglycemia. It is frequently overlooked and may result in unnecessary investigations and management. We describe a 14-year-old girl with long-standing poorly controlled type 1 diabetes mellitus (HbA1c 15.1%) who, the week following recovery from severe diabetic ketoacidosis and a rapid increase in insulin therapy, experienced acute edema and gained seven kilos of weight over the next week. Clinical examination and laboratory testing ruled out infectious, cardiac, hepatic, or intrinsic renal etiologies of edema. Insulin-induced edema was diagnosed because of the temporal relationship between insulin regimen escalation, rapid fluid weight gain, and resolution without any intervention. The edema resolved completely without the administration of diuretics or cessation of insulin therapy. This case highlights the importance of maintaining a high level of clinical suspicion for insulin-induced edema, which is a benign and self-limited diagnosis of exclusion in children and adolescents who experience rapid metabolic recovery after prolonged hyperglycemia, to prevent unnecessary interventions and unsafe reductions in insulin therapy.

## Introduction

Type 1 diabetes mellitus (T1DM) is best managed with insulin, and early, persistent glycemic control is imperative to avoid both short-term metabolic decompensation and long-term microvascular and macrovascular complications. Although there have been substantial improvements in insulin types and delivery, adolescents with T1DM still experience significant difficulties with adherence, psychosocial adjustment, and blood glucose fluctuation [1]. According to international clinical guidelines from the American Diabetes Association and the International Society for Pediatric and Adolescent Diabetes [1,2], poor metabolic control in adolescence is strongly linked to a higher risk of diabetic ketoacidosis (DKA), early nephropathy, hypertension, and recurrent hypoglycemia, especially in patients who do not use insulin regularly or examine their glucose levels often.

Insulin-induced edema is a rare but known clinical complication associated with insulin therapy, usually presenting after administration of insulin or rapid up-titration of doses in patients with prolonged inadequately controlled hyperglycemia. Although first reported more than seven decades ago during early insulin therapy, the condition remains rare in current clinical practice, particularly in children and adolescents [3]. The exact prevalence is not known, and it is likely underreported because of a lack of awareness and the frequent mistaken diagnosis as cardiovascular, renal, hepatic, or infectious disease. Insulin edema is commonly observed in patients recovering from profound metabolic catabolism (e.g., new-onset diabetes or DKA) when rapid insulin repletion occurs, causing sudden changes in fluid and electrolyte balance [3,4].

The underlying mechanism of insulin-induced edema is complex. Insulin directly inhibits natriuresis in the renal tubules, augmenting sodium reabsorption in the proximal nephron and activating the renin-angiotensin-aldosterone system to facilitate sodium and water reabsorption [4,5]. Concurrently, insulin increases capillary permeability and enhances intracellular glucose uptake, thereby generating oscillatory osmotic gradients that drive interstitial fluid redistribution and associated edema. Other contributory mechanisms include rapid glycogen synthesis with associated water binding, suppression of glucagon-induced natriuresis, and loss of plasma oncotic pressure during the post-catabolic refeeding period [4].

In clinical practice, insulin-induced edema can manifest as focal swelling at injection or sensor sites or generalized pitting edema of the face, feet, and superficial tissues. Life-threatening complications, including pleural effusions, ascites, and cardiopulmonary failure, have been described but are rare [3-5]. It is a diagnosis of exclusion and should be characterized by careful evaluation for heart failure, nephrotic syndrome, acute kidney injury, hepatocellular dysfunction, venous thrombosis, and soft tissue infection. Furthermore, the edema associated with insulin-induced edema is generally transient and self-limiting, with spontaneous resolution occurring within days to weeks, even if insulin therapy is not discontinued [3,4].

Pediatric cases of insulin-induced edema are few and are largely reported as individual case reports and limited microseries. Adolescents with prolonged poor glycemic control seem to be most at risk, particularly after rapid correction of hyperglycemia in DKA or aggressive insulin intensification [5]. Failure to identify this phenomenon can lead to inappropriate diuretic treatment and undue cardiac and renal investigations, as well as precipitous reduction or even cessation of insulin therapy, thus potentially increasing the rate of recurrent DKA and long-term complications [3-5].

We present the case of a teenager with extremely poorly controlled T1DM who developed insulin-induced regional edema and experienced an acute 7 kg weight gain following the rapid correction of metabolic derangements. This case emphasizes the diagnostic difficulties, the pathophysiology that leads to disease severity, and the importance of early recognition of this benign but extremely distressing complication.

## Case presentation

We present the case of a 14-year-old female with T1DM diagnosed at 10 years of age who presented with short-duration localized swelling following augmentation of her insulin therapy. Her diabetes had been severely uncontrolled, with her glycated hemoglobin level being 15.1%. She was treated with basal-bolus insulin therapy, using rapid-acting insulin before each meal. Glycemic monitoring was also inconsistent due to placement issues with the continuous glucose monitor (CGM) and the fact that blood glucose levels were measured only once or twice daily.

The patient was hospitalized for a week with severe DKA after missing her basal insulin for two weeks and using only rapid-acting insulin inappropriately once daily. Upon discharge, she underwent an aggressive insulin therapy regimen to normalize her long-standing hyperglycemia. During this time, she temporarily switched to the ultra-rapid-acting insulin as a result of continuous hyperglycemia. She subsequently incurred recurrent hypoglycemia related to insulin overcorrection and carbohydrate mismatching. Her mother, concerned for hypoglycemia, temporarily kept her glucose targets at deliberately high levels. The patient was later switched back to regular rapid-acting insulin.

During this post-catabolic recovery stage, she had a sudden-onset weight gain of approximately 7 kg over one week, with no concomitant increase in dietary caloric intake or reduction in physical activity, suggesting that the gain was primarily due to acute fluid retention rather than fat accretion. She had bilateral lower limb pitting pedal edema. A cardiac examination revealed normal heart sounds without murmurs, and the lungs were clear. There was no clinically detectable ascites or hepatosplenomegaly, and the abdominal examination was unremarkable.

At the time of edema diagnosis, capillary blood ketones were normal, acid-base status was normal, serum creatinine was within the normal range, and liver transaminases were normal. There was minimal proteinuria. Moreover, there are no clinical or laboratory features to suggest heart failure, nephrotic syndrome, acute kidney injury, hepatic dysfunction, or soft-tissue infection (Table 1). 

**Table 1:** Laboratory findings at presentation Abbreviations: HbA1c, glycated hemoglobin; HCO₃, bicarbonate; ESR, erythrocyte sedimentation rate; MCV, mean corpuscular volume; MCH, mean corpuscular hemoglobin; MCHC, mean corpuscular hemoglobin concentration; RDW, red cell distribution width; HPF, high-power field

Parameter	Result	Reference range
HbA1c	15.1%	<5.7%
Random blood glucose	117 mg/dL	72–112 mg/dL
Sodium	136 mmol/L	136–145 mmol/L
Potassium	4.4 mmol/L	3.8–5.5 mmol/L
Chloride	102 mmol/L	101–107 mmol/L
Bicarbonate (HCO₃)	21.5 mmol/L	17–26 mmol/L
Urea	33 mg/dL	12–40 mg/dL
Creatinine	0.53 mg/dL	0.5–0.9 mg/dL
Calcium	9.7 mg/dL	9.3–10.6 mg/dL
Phosphate	4.6 mg/dL	3.1–5.3 mg/dL
Magnesium	1.67 mg/dL	1.7–2.2 mg/dL
Albumin	4.0 g/dL	4.3–5.3 g/dL
Total protein	7.2 g/dL	6.3–7.8 g/dL
C-reactive protein	1.3 mg/L	0.4–2.0 mg/L
ESR	19 mm/hr	0–20 mm/hr
Hemoglobin	10.9 g/dL	12.0–15.0 g/dL
Hematocrit	35.2 %	36.0–46.0%
MCV	83.6 fL	77–95 fL
MCH	25.9 pg	27–32 pg
MCHC	31.0 g/dL	31.5–34.5 g/dL
RDW	14.9 %	11.5–14.0%
White blood cells	6.3 × 10³/µL	3.6–11.0 × 10³/µL
Platelets	531 × 10³/µL	150–410 × 10³/µL
Urine protein (dipstick)	1+	Negative
Urine glucose	3+	Negative
Specific gravity	1.033	1.002–1.030
Urine protein (random)	30.6 mg/dL	<15.0 mg/dL
Urine creatinine	161.0 mg/dL	—
Protein/creatinine ratio	190 mg/g	<150 mg/g
RBCs	0–2 /HPF	0–2 /HPF
WBCs	0–5 /HPF	0–5 /HPF

**Figure 1:**
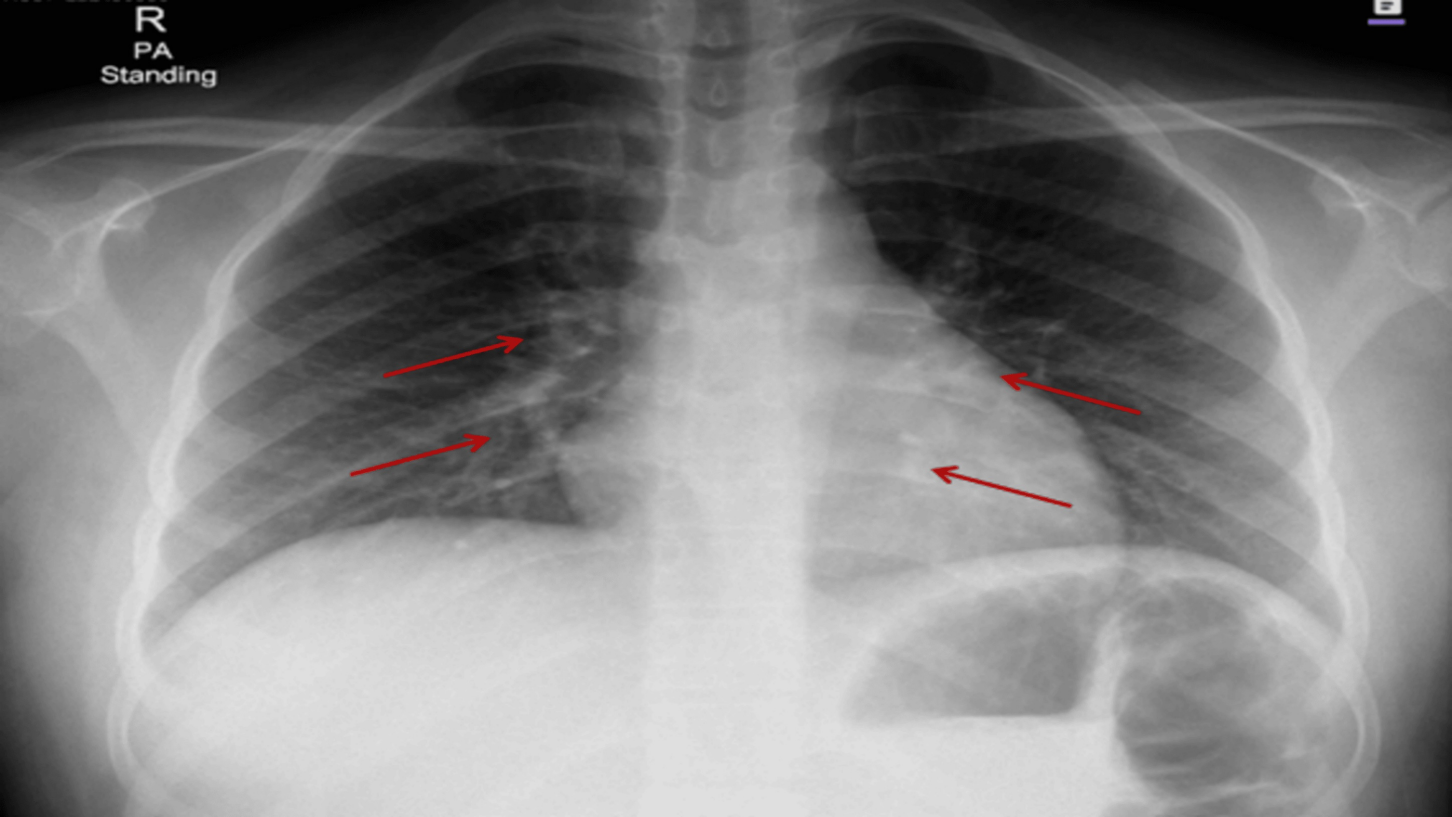
Posteroanterior chest radiograph showing prominent bilateral perihilar bronchovascular markings (arrowed) with a normal cardiac silhouette and no radiographic evidence of pleural effusion.

A chest X-ray (posteroanterior view) was performed to rule out pleural effusion, which revealed bilateral prominent perihilar bronchovascular markings with a normal cardiac shadow and no evidence of pleural effusion or lobar consolidation (Figure 1). 

Given the close temporal relationship between the rapid reinitiation and uptitration of insulin after severe DKA, the patient's presentation of an intense 7 kg acute weight gain over one week, along with negative cardiac, renal, and hepatic workup results, led to a diagnosis of insulin-induced edema. The patient was supported conservatively, with reassurance, insulin continued at the same dose without reduction, proper injection-site rotation and sensor placement reminders, and follow-up care as an outpatient. No diuretics were given. The edema spontaneously resolved within a one-month interval without recurrence.

## Discussion

Insulin-induced edema is a rare, benign, but underrecognized adverse effect of insulin treatment; it was first recognized soon after the introduction of insulin into clinical practice and remains reported almost exclusively in isolated case reports and small case series [3-5]. It usually presents after the onset or sudden intensification of insulin therapy, particularly in people with a period of extended uncontrolled hyperglycemia or recovering from a catabolic state, as is the case post-DKA [3,4]. The current patient presented with all the classic predisposing factors of insulin-induced edema: long-standing severe hyperglycemia, sudden reintroduction, an increased dose of insulin following a prolonged period of omission, a recent episode of severe DKA, and rapid metabolic improvement.

The exact etiology of insulin edema is multifactorial and not yet well understood. Insulin has an intense antinatriuretic action, which is mediated by stimulating the reabsorption of sodium in the proximal renal tubules, which occurs due to an enhancement of sodium-hydrogen exchanger activity and Na⁺/K⁺-ATPase function [4]. Concomitantly, insulin stimulates the renin-angiotensin-aldosterone system, thereby promoting renal sodium and water reabsorption [4,5]. Insulin also increases capillary permeability and facilitates the movement of glucose into cells, with a rapid gradient shift causing transient fluid shifts in the interstitium. In the post-catabolic phase, newly formed glycogen binds water at approximately a 1:3 ratio and acutely increases intravascular volume [4].

This patient demonstrated one definite feature of insulin-induced edema: a 7 kg weight gain in one week, which is not physiologically possible to accumulate as adipose tissue and suggests acute fluid retention. Rapid weight gain has also been described in both adult and pediatric forms of insulin edema, sometimes representing the first clinical hint of its diagnosis [5]. 

Insulin-induced edema usually occurs as dependent or generalized edema; however, localized reactions at injection or sensor insertion sites have been increasingly reported with modern insulin delivery systems and continuous glucose monitoring devices [6]. The diagnosis of insulin edema remains a case of exclusion. In this patient, no cardiovascular abnormalities were identified on examination, and oxygen saturation was satisfactory. Renal function was maintained, with no signs of acute kidney injury or nephrotic syndrome, and liver synthetic and enzymatic functions remained normal. There were no findings of venous thrombosis or systemic inflammatory response. The close time interval of insulin intensification, swift metabolic correction post DKA, and fast weight gain with subsequent development of edema, only to resolve spontaneously under conservative treatment, points strongly towards insulin-induced edema being the leading diagnosis.

Treatment of insulin-induced edema is essentially conservative. Overseas experience always suggests that mild-to-moderate cases can maintain insulin without adjustments in dosage [7]. Salt restriction may be used in the presence of generalized edema, and diuretics are reserved for severe or symptomatic fluid overload. It is critical to note that the discontinuation or inappropriate reduction of insulin dosing is contraindicated owing to continued metabolic instability and because it significantly increases the risk for recurrent DKA. In children, reassuring the patient and family is key, given that parental fear of hypoglycemia often fuels insulin underdosing, leading to severe hyperglycemia. In this case, simple conservative management resulted in spontaneous complete resolution of edema and also avoided recurrence.

This case report adds to the already established psychosocial aspect of unstable puberty diabetes by including hypoglycemia fear, insulin omission, and parental anxiety. All these factors together resulted in extreme glycemic variability and repeated DKA. Diagnosis of insulin edema not only avoids unnecessary investigations and treatment, but it also presents an opportunity to provide targeted diabetes education, put in place psychological support, and encourage adherence with safe injection practice, which is key to long-term metabolic stability.

## Conclusions

Insulin-induced edema is an uncommon but significant condition in patients receiving rapid insulin therapy escalation, especially after prolonged neglected hyperglycemia and immediate recovery from diabetic ketoacidosis. The diagnostic dilemma in this case is typical of etiologically misplaced acute weight gain and localized edema that followed very rapid insulin reinitiation and rapid metabolic correction in an adolescent with volatile T1DM. Recognizing insulin-induced edema as a benign, self-limited diagnosis of exclusion is crucial to avoid unwarranted testing, antimuscarinic or diuretic therapy, and, most importantly, harmful reduction or cessation of insulin. Early diagnosis, caregiver education and reassurance, and ongoing insulin therapy with frequent metabolic follow-up still form the basis of care and are essential to avoid repeated episodes of DKA and long-term microvascular sequelae.
